# Maximal dynamic inspiratory pressure: S-Index prediction values and diagnosis accuracy

**DOI:** 10.36416/1806-3756/e20240409

**Published:** 2025-09-22

**Authors:** Vitor Costa Souza, Maria Fernanda Lima Souza Saldanha, Eloara Vieira Machado Ferreira, Luiz Eduardo Nery, Priscila Cristina de Abreu Sperandio

**Affiliations:** 1. Função Pulmonar e Fisiologia Clínica do Exercício, Divisão de Pneumologia, Universidade Federal de São Paulo, São Paulo (SP) Brasil.

**Keywords:** Maximal respiratory pressures, Muscle strength, Respiratory muscles

## Abstract

**Objective:**

To establish reference values and prediction equations for the strength index (S-Index), in order to meet the growing demand for clinical application and diagnostic understanding of maximal dynamic inspiratory pressure.

**Methods:**

This was a prospective study of 120 healthy subjects between 18 and 80 years of age. The S-Index, measured from RV to TLC after at least eight reproducible maximal maneuvers with a < 10% difference, was obtained. The MIP was also measured, and differences between S-Index and MIP values were analyzed. A multiple linear regression model estimating the S-Index value was based on clinically significant independent variables. For model cross-validation and diagnostic accuracy, we used a separate sample of COVID-19 survivors to compare the observed and predicted S-Index values.

**Results:**

The S-Index strongly correlated with the FEV_1_ and FVC. However, sex, age, weight, and height retained their significance in all final models, collectively explaining 62% of the variation in the observed values. The performance of the prediction equation was satisfactory in suggesting differences between COVID-19 survivors with an MIP < 80 cmH_2_O and those with an MIP ≥ 80 cmH_2_O. For both sexes, the S-Index exhibited the potential for ruling out, rather than confirming, inspiratory muscle weakness. If below the lower limit of normal, further evaluation is important, especially in men.

**Conclusions:**

To our knowledge, this is the first set of reference equations for the S-Index based on a healthy adult population across various age groups in Brazil. Its potential as an adjunct index in evaluating inspiratory muscle strength was also explored for the first time.

## INTRODUCTION

In assessing the respiratory system, the inspiratory muscles serve as the primary pump for effective ventilation.^1^ In clinical settings, the maximal static inspiratory pressure (MIP) has traditionally been measured to estimate inspiratory muscle strength, given that gold-standard invasive techniques are often impractical to implement outside of research environments.^2^ However, advances in device technology have introduced alternative methods for assessing inspiratory muscle strength, such as the maximal dynamic pressure, the application of which has now expanded beyond healthy individuals^3^ ^-^ ^6^ to include patients with heart failure,^7^ stroke survivors,^8^ and children with asthma.^9^ For that test, the subject performs a forceful inspiration, from RV to TLC, through an open valve, while the dynamic inspiratory pressure is plotted continuously for each lung volume during inspiration, creating a timeline.^10^ The peak value is known as the strength index (S-Index), which is believed to reveal valuable information about the inspiratory muscle capacity to generate volume and its impact on overall performance in patients and athletes.^11^

The S-Index is an adjunct index provided by an inspiratory training device (POWERbreathe KH2; HaB International, Southam, UK); it has been well established that the S-Index cannot replace the MIP.^10^ ^,^ ^11^ Amid the growing popularity of the S-Index,^5^ some studies have erroneously treated this dynamic parameter as equivalent to the MIP.^8^ ^,^ ^12^ ^,^ ^13^ During the COVID-19 outbreak, more patients underwent evaluation of inspiratory muscle strength due to the acute and long-term effects of the disease, which extend beyond the respiratory system.^14^ This underscores the importance of clearly distinguishing the S-Index from the MIP to ensure accurate assessment and interpretation.

The S-Index and the MIP are different technical measurements and do not represent the same physiological information or muscle recruitment, because the S-Index, unlike the MIP, is flow-dependent.^7^ On that basis, the S-Index is believed to be a functional parameter, mimicking the normal, resistance-free contraction of inspiratory muscles.^5^ ^,^ ^11^ Despite studies demonstrating a strong correlation between the S-Index and the MIP, given that both measure the same property, there is wide variability between their values.^10^ ^,^ ^11^ This could be related to static and dynamic contractions, reflecting the contrast between the generality of the MIP and the specificity of the S-Index for the same task.^15^

It has long been recognized that individual measurements hold limited significance unless they can be compared to a reference value.^16^ A 2021 study proposed a set of equations predicting the S-Index on the basis of a sample of 92 healthy, fit elderly volunteers, mostly women, although those equations are not generalizable to other age groups.^6^ Despite some progress, there have been, to our knowledge, no studies providing reference values for the S-Index in differing age groups.

The aim of this study was to generate S-Index prediction equations for males and females, as well as to understand their accuracy in determining inspiratory muscle weakness, using MIP as a reference. A separate sample of COVID-19 survivors was used in order to compare the observed S-Index values with those predicted by the equations.

## METHODS

### Study design and participants

This was a cross-sectional study in which we evaluated healthy subjects between 18 and 80 years of age. Participants were recruited through verbal, online, and printed invitations during the 2018-2020 period. The inclusion criteria were being a nonsmoker and having a BMI ≤ 30 kg/m^2^. Individuals with abnormal spirometry results-values under the lower limit of normal (LLN)-were excluded, as were those who used illicit drugs, those who were athletes, those with self-reported respiratory or heart disease, neuromuscular or thoracic orthopedic conditions, history of thoracic or abdominal surgery, or pregnancy, and those who were incapable of understanding the proposed tests, as well as those with inspiratory muscle weakness, according to the predictive values published previously.^17^ Subsequently, we analyzed data from COVID-19 survivors, collected at a later stage, to compare the observed S-Index values with the values predicted by the equations derived in the present study.

Although a formal sample size calculation was not performed, this convenience sample was intended to offer preliminary insights into the differences between the MIP and S-Index and to develop a prediction equation for the latter. The study was approved by the Research Ethics Committee of the Federal University of São Paulo (Reference no. 2.410.123/2017), and all participants gave written informed consent.

### Procedures

Data related to demographics, current health status, past illnesses, history of surgery, and smoking habits were obtained. To evaluate physical activity levels, we used the modified Baecke Index questionnaire.^18^ To measure body weight and height, we used a calibrated scale and a stadiometer with subjects in light clothing and standing barefoot. 

None of the subjects were habituated to the test or had ever undergone this assessment. The protocol was explained, and all techniques were demonstrated before each of the tests, all of which were performed by the same evaluator. Volunteers were submitted to a warm-up and familiarization stage.^3^ To qualify for the familiarization stage, the individuals were evaluated by a trained physiotherapist to determine whether they could follow the previously established guidelines. Volunteers who failed the familiarization stage were excluded from the study.

### Pulmonary function

Pulmonary function was assessed with a spirometer (CareFusion Microloop; Becton, Dickinson and Company, Franklin Lakes, NJ, USA), in accordance with the recommendations of the American Thoracic Society.^19^ We selected the FVC (in L) and FEV_1_ (in L) obtained after at least three forceful expiration maneuvers, subsequently comparing those with the values predicted for the population of healthy adults in Brazil.^20^

### Inspiratory muscle strength assessment

All tests involved the use of the same handheld POWERBreathe device, which was connected to a computer running software specific to the device (BREATHELINK; HaB International). A rubber-flanged mouthpiece originally designed for the device was used. 

The mouth MIP (in cmH_2_O) was obtained after at least five acceptable maximal maneuvers (forceful inspirations from RV to TLC), with three of them having a difference of less than 10%. An inspiratory effort of at least 1.5 s was maintained so that a plateau pressure sustained for 1 s could be recorded. Subjects were advised to rest for one minute between efforts.^21^ ^,^ ^22^

After the MIP value had been obtained, the dynamic inspiratory pressure was determined by identifying the greatest S-Index value (cmH_2_O) in maneuvers performed from RV to TLC. At least eight maximal maneuvers that were reproducible (with a < 10% difference) were performed to avoid interpretive errors associated with learning effects.^3^ During each S-Index test, flow (L/s) and volume (L) are also provided and recorded for analysis. The operational differences between static and dynamic assessments of inspiratory muscle strength, in terms of the mechanisms of the device valve, are shown in the supplementary online videos.

The highest value from an acceptable inspiratory curve was selected unless it was reached in the final maneuver.^21^ ^,^ ^22^ Tests were repeated after 30 min of rest to confirm the results obtained. Each volunteer then completed a minimum of ten MIP maneuvers and sixteen S-Index maneuvers.

As illustrated in Figure S1, subjects remained seated with their back resting against the chair back, wearing a nose clip, with their lips tightly closed around the mouthpiece to avoid air leaks. The maximal effort was encouraged in the form of standardized vigorous verbal stimulation, and closer attention was given to avoiding the use of facial muscles and compensatory movements of the head and trunk.^21^ ^,^ ^22^

### Comparison among COVID-19 survivors

We used data from COVID-19 survivors to compare observed and predicted S-Index values, employing the equations derived in the present study. Using a different validation sample yields a measure of the future performance of the model that is more unbiased and less optimistic, because the magnitude of the residual is less likely to be impacted.^23^ The same protocol for evaluating inspiratory muscle strength was used in the collection of these data, which was performed by the same investigators. Subjects were recruited from the post-COVID-19 outpatient clinic of São Paulo Hospital, operated by the Federal University of São Paulo in the city of São Paulo, Brazil. The protocol mentioned is part of a larger study assessing respiratory muscle strength, pulmonary function, exercise capacity, and dyspnea. It was approved by the Institutional Review Board of São Paulo Hospital (Reference no. 4.346.971, from 19 October 2020). Other papers analyzing this sample of COVID-19 survivors have been published.^24^ ^,^ ^25^ From that large sample of COVID-19 survivors (N = 361), we analyzed the diagnostic accuracy of the S-Index as a surrogate parameter for identifying inspiratory muscle weakness. To determine the sensitivity, specificity, positive predictive value (PPV), and negative predictive value (NPV), we used the LLN for the S-Index and a cutoff of 80 cmH_2_O for the MIP. The age-specific LLN was calculated by using z-scores, with the following formula^26^:

LLN=age−specificmean−(1.645×standarderroroftheestimate)

### Statistical analysis

A visual curve analysis was performed before the final inclusion of the data, which were subsequently analyzed with the IBM SPSS Statistics software package, version 21.0 (IBM Corp., Armonk, NY, USA) and GraphPad Prism, version 9 (GraphPad Software, Inc., San Diego, CA, USA). Normality tests and visual data inspection revealed variable normality distribution. The data were stratified by sex and age group (20-39, 40-59, and 60-80 years), and the descriptive statistics were compared by using the Student’s t-test. After correlating MIP values against the S-Index, the Bland-Altman method was employed to investigate the agreement between them, for all sample sizes and for males and females separately.^27^

To investigate the relationships among them using demographic, anthropometric, and clinical data, we performed correlation analyses. Using multiple linear regression with least-squares minimization, we included independent variables with significant clinical relevance and statistical significance in a model to estimate the S-Index value, with sex and age serving as adjustment factors. Variables were included in order of decreasing correlation coefficient, and the F probability was used to add or remove variables.

For all data, the coefficient of determination (R^2^) is reported with the residual standard error, the equation of the regression line, and the partial coefficients with their standard errors. For all analyses, values of p < 0.05 were considered statistically significant. 

## RESULTS

A total of 153 subjects were recruited. Of those, 15 were considered ineligible. Of the 138 remaining individuals, 18 were excluded: 15 because they presented with altered pulmonary function; and 3 because they were unable to perform respiratory tests properly. Therefore, the final sample comprised 120 individuals (50 men and 70 women), stratified by age into three groups (Figure S2).

**Figure 1:**
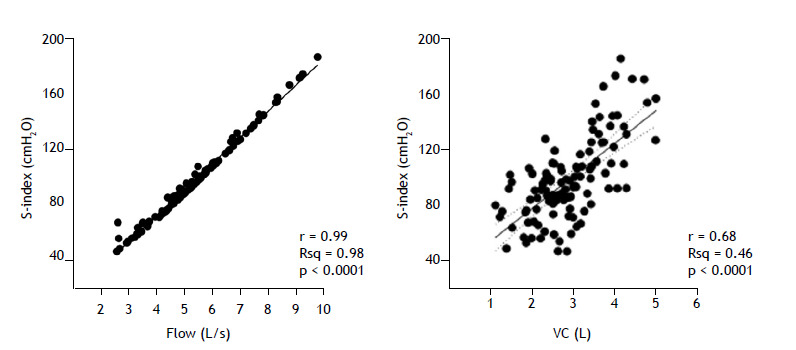
The graph on the left shows the positive correlation between the strength index (S-Index) and inspiratory flow-*S-Index* = (18.78 × *flow*) − 3.85, estimated R^2^ (Rsq): 0.98, p < 0.0001. The graph on the right shows the correlation between the S-Index and the inspiratory VC for the sample as a whole-*VC* = (0.02 × *S-Index*) + 0.99 (standard error of the estimate: 0.63), Rsq: 0.46, p < 0.0001. Regression lines are presented with the corresponding 95% confidence intervals.

Because the S-Index is a flow-dependent measure derived from a non-occluded valve, it is important to determine the correlation between the S-Index and inspiratory flow; Figure 1 illustrates their perfect positive correlation (r = 0.99; p < 0.0001). The figure also shows a moderate positive correlation between the S-Index and the inspiratory VC generated during each maneuver from RV to TLC (r = 0.68; p < 0.0001), which was found to decrease with age (Figure S3).

**Figure 2:**
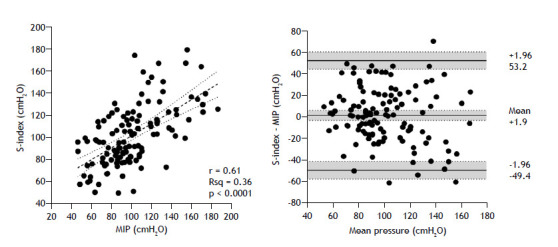
On the left, the strength index (S-Index) values compared with the MIP values in 120 healthy subjects. Linear regression analysis generated an equation-*S-Index* = (0.67 × *MIP*) + 30 (standard error of the estimate: 24), estimated R^2^ (Rsq): 0.36. On the right, a Bland-Altman plot of differences between the S-Index and MIP. Shaded areas represent 95% confidence intervals.

Baseline characteristics and parameters are summarized in Table 1. For the total sample size, the mean MIP value showed no significant difference in comparison with the mean S-Index (102 ± 27 vs. 100 ± 31 cmH_2_O, p = 0.43), a finding that was consistent across all age groups. Figure 2 shows a moderate correlation between the S-Index and the MIP (r = 0.61, p < 0.0001), and a Bland-Altman plot reveals a narrow, not significant bias (+1.9 cmH_2_O, 95% CI: −2.8 to 6.6) with large variability (range, −49.4 to 53.2 cmH_2_O).

**Table 1:** Anthropometric, spirometry, and inspiratory muscle strength data, by sex and age group.^a^

Variable	Men			Women		
	(n = 50)			(n = 70)		
	Age group (years)			Age group (years)		
	20-39	40-59	60-80	20-39	40-59	60-80
	(n = 27)	(n = 15)	(n = 8)	(n = 26)	(n = 23)	(n = 21)
Anthropometric data						
Age, years	28 ± 6	49 ± 7	70 ± 7	29 ± 5	52 ± 6	73 ± 16
Height, cm	179 ± 8	170 ± 5	170 ± 6	164 ± 5	160 ± 7	157 ± 6
Weight, kg	78 ± 13	71 ± 8	74 ± 9	60 ± 7	70 ± 6	60 ± 8
BMI, kg/cm^2^	24 ± 3	24 ± 2	25 ± 3	22 ± 3	25 ± 3	24 ± 3
Physical activity score	6 ± 4	6 ± 3	7 ± 2	6 ± 1	6 ± 1	7 ± 2
Spirometry parameters						
FEV_1_, L	3.6 ± 1.6	3.2 ± 1	3.1 ± 0.5	3.1 ± 0.3	2.6 ± 0.3	2.0 ± 0.4
FEV_1_, % of predicted	81.8 ± 36.1	88.7 ± 27.4	99.2 ± 11.1	94.6 ± 9.7	100.1 ± 9.7	99.6 ± 13.5
FVC, L	4.2 ± 1.9	3.9 ± 1.1	3.9 ± 0.7	3.4 ± 0.4	3.0 ± 0.4	2.4 ± 0.5
FVC, % of predicted	80.0 ± 35.2	89.6 ± 26.3	96.2 ± 15.6	92.4 ± 10.3	96.3 ± 9.1	89.8 ± 11.5
FEV_1_/FVC ratio	85.7 ± 38.6	76.8 ± 21.9	80.7 ± 9.9	90.5 ± 6.4	87.6 ± 6.6	86.9 ± 9.0
Inspiratory muscle strength						
MIP, cmH_2_O	127 ± 30*	114 ± 17*	109 ± 25*	100 ± 16	89 ± 17	75 ± 15
S-Index, cmH_2_O	137 ± 28*	113 ± 21*	105 ± 23*	92 ± 14	80 ± 17	73 ± 16
MIP − S-Index	−10 ± 33	1 ± 27	4 ± 27	8 ± 24	9 ± 21	2 ± 22
MIP − S-Index, p-value	0.12	0.86	0.62	0.10	0.51	0.7

S-Index: strength index. ^a^All data, except statistical data, expressed as mean ± SD. *p < 0.01 vs. women (t-test).

The increasing variance toward higher values implies troubled agreement and an equivalence problem. In a sub-analysis considering sex, the bias between the S-Index and the MIP was significant for the women (6.4 cmH_2_O, 95% CI: 2.4 to 10.3) but not for the men (−4.4 cmH_2_O, 95% CI: −1.0 to 9.8).

Males had higher S-Index values than did their age-matched female counterparts. The S-Index decreased with advancing age, which presented a significant negative effect (r = −0.42, p < 0.0001 for men and r = −0.44, p < 0.0001 for women), as illustrated in Table 2 and Figure S4. As depicted in Figure S5, height had the strongest correlation with the S-Index (r = 0.72, p < 0.0001). In addition, FEV_1_ and FVC showed significant positive correlations with the S-Index.

**Table 2:** Correlation matrix.^a^

Variable	Variable						
	Age	Weight	Height	BMI	FEV_1_	FVC	S-Index
Age	1						
Weight	−0.14	1					
Height	−0.46*	0.69*	1				
BMI	0.26*	0.67*	−0.06	1			
FEV_1_	−0.44*	0.31*	0.46*	−0.03	1		
FVC	−0.35*	0.38*	0.50*	0.02	0.96*	1	
S-Index	−0.45*	0.59*	0.72*	0.07	0.41*	0.43*	1

S-Index: strength index. ^a^Values represent the Pearson correlation coefficient between variables. *p < 0.01.

In the multiple linear regression analysis, age, height, and weight remained in all final models, collectively explaining 62% of the S-Index variation (Table 3). Spirometric variables lost their independent predictive power after those basic anthropometric variables were considered in the multiple regression, with a lower adjusted R^2^ and R^2^ change when compared with the previous model of prediction. After the predicted residual sum of squares method was applied to the model equations, the original values of R^2^ and standard error of the estimate were found to have only a mild effect.

**Table 3:** Prediction equation for the strength index in healthy subjects.

	Constant	Age (years)	Weight (kg)	Height (cm)	R^2^	RSE
		CE ± SEE	CE ± SEE	CE ± SEE		
S-Index (cmH_2_O)						
Male	− 32.3	−0.39 ± 0.11	0.47 ± 0.22	0.79 ± 0.32	0.62	19.27
Female	− 54.0	−0.39 ± 0.11	0.47 ± 0.22	0.79 ± 0.32	0.62	19.27

S-Index: strength index; CE: coefficient estimate; SEE: standard error of the estimate; R^2^: coefficient of determination; and RSE: residual standard error.

Table S1 shows the mean difference between the expected and observed S-Index values among the 361 COVID-19 survivors evaluated. For men and women with an MIP ≥ 80 cmH_2_O, the observed S-Index values closely matched the predicted values, indicating good agreement. Conversely, in patients with an MIP < 80 cmH_2_O, the observed S-Index values were significantly lower than were the predicted values, statistically and clinically, demonstrating the ability of the equation to distinguish among different clinical profiles in terms of inspiratory muscle performance.

For each patient, we analyzed the LLN derived from the S-Index z-score and classified it by the presence of inspiratory muscle weakness, using the same MIP cutoff (Table S2). The sensitivity of the S-Index for detecting true inspiratory muscle weakness was 76% for men and 18% for women. In terms of specificity, the S-Index correctly identified patients without muscle weakness at a rate of 75% for men and 95% for women. In our study sample, the PPV-the probability of having inspiratory muscle weakness when the result was positive-was 31% for men and 75% for women. The NPV was 95% for men and 64% for women. 

## DISCUSSION

To our knowledge, this is the first study to provide a set of S-Index prediction equations derived from healthy adults of different ages by using a standardized methodology according to established guidelines.^21^ ^,^ ^22^ One study published S-Index reference equations specific to an elderly population (mean age of 72 ± 5 years), based on 92 subjects, mostly women, incorporating FEV_1_, six-minute walk distance, age, and height in the prediction models.^6^ Those equations incorporate tests that are more sophisticated as independent variables to predict the S-Index, transforming a straightforward task into a complex investigation. By using simple demographics and anthropometric data in the prediction model, we increase the likelihood of achieving large populations, generalizing the applicability from large hospitals and research centers to home-based care.^28^ ^,^ ^29^

Given the aspects mentioned above, our comparison is limited. When tested on a sample of 361 COVID-19 survivors to evaluate prediction accuracy, the S-Index showed a small mean difference of only 5 cmH_2_O between the expected and observed values (95% of the predicted value) among women without inspiratory muscle weakness. Although statistically significant, that difference is unlikely to have a notable clinical impact when studying subjects without muscle weakness. For the 173 men without muscle weakness, a significant difference of 13 cmH_2_O was observed between expected and observed values. However, when looking at the percentage of the predicted value, reaching a mean of 90% is generally considered a good indication of normality in clinical terms. 

The distinction between normality and disease becomes more apparent when COVID-19 survivors with inspiratory muscle weakness, as suggested by lower MIP, are examined. In our sample of such individuals, the difference between the expected and observed values for men was significant at 36 cmH_2_O, representing 70% of the predicted value, and at 14 cmH_2_O for women, corresponding to 84% of the predicted value. Patients with muscle impairment can produce a lower mean inspiratory flow,^30^ which could lead to a markedly lower S-Index than that observed in those without it.

One original finding of this study is the capability of the S-Index to serve as a potential surrogate parameter for detecting impaired inspiratory muscle strength. Although we have used MIP values as the reference standard, previous studies, acknowledging that MIP is not the gold standard for diagnosing specific diaphragm weaknesses,^31^ have demonstrated its potential in various applications,^32^ such as in the early detection of intensive care unit-acquired weakness, for which it has been shown to have 88% sensitivity and 76% specificity.^33^

Among the COVID-19 survivors evaluated in the present study, the S-Index exhibited greater potential for ruling out inspiratory muscle weakness than for confirming it, regardless of the sex of the patient. When the S-Index falls below the LLN, further evaluation is warranted, especially in men. When the result was positive, the test was more reliable in confirming weakness in women than in men, whereas, when the result was negative, it was better at excluding weakness in men than in women.

The choice of using a cutoff pressure of 80 cmH_2_O to categorize groups is a limitation that may have affected our results. It is generally agreed that values above 80 cmH_2_O are not indicative of significant weakness.^21^ The use of the 80 cmH_2_O cutoff risks misdiagnosis this condition in women, given that a recent study involving 610 healthy subjects in Europe suggested that the threshold is lower (62 cmH_2_O) for women.^34^ After all, a fixed cutoff fails to account for variations in sex and age, which significantly influence strength values.

To our knowledge, there have been no studies evaluating the S-Index in different age groups. That is why we partitioned our comparison. In a study of 43 healthy adults with a mean age of 37 ± 9 years,^3^ the S-Index for the inspiratory muscle warm-up group was 123 cmH_2_O, with no sex-based differences.^3^ In contrast, we found a significant difference between males and females in the 20- to 39-year age group (137 ± 28 cmH_2_O vs. 92 ± 14 cmH_2_O). Similarly, a recent survey of 597 young athletes with a mean age of 21.4-22.0 years reported a mean S-Index of 145 ± 30 cmH_2_O for the men, compared with 101 ± 28 cmH_2_O for the women.^5^ This highlights notable sex-based differences in S-Index values. For older age groups, our S-Index values showed significant variation in comparison with those published in a previous study.^6^

Among the variables evaluated in the present study, height exhibited the strongest predictive ability. In the literature (old and new), it has consistently been shown that there is a correlation between height and lung capacity, with taller individuals generally having larger lungs and therefore a greater ability to store air.^35^ ^-^ ^37^ During a dynamic strength test, the open valve enables passage of inspiratory volume, detecting flow and resulting in the S-Index pressure. That relationship is further supported by the fact that the S-Index correlates significantly with the inspiratory vital capacity and with spirometric variables.

An additional aim of the present study was to analyze the differences between the MIP and S-Index values. Although both assess inspiratory muscle strength and demonstrate a moderate correlation, they show significant variability. That variability highlights their distinct mechanisms and emphasizes the idea that they should not be used interchangeably. This is confirmed by the large standard deviations compared to the small mean values, along with notable discrepancies in the agreement analysis, which align with previous studies reporting differences greater than 50 cmH_2_O.^7^ ^,^ ^10^ ^,^ ^11^ This significant variability introduces a level of unpredictability, making it unwise to use bias as a correction factor to interchange values between the two measures.

Our study has some strengths. To better understand the behavior of the S-Index as a clinical parameter, we made efforts to collect data from individuals of different ages and sexes, as well as with different BMIs, activity levels, and exercise habits, representing the general population. In contrast with previous studies, which used three, five, or ten maximal inspiratory maneuvers, we conducted a minimum of sixteen assessments to obtain reliable maximum S-Index values preceding inspiratory muscle warm-up, as previously described.^3^

Our study also has some notable limitations, including the fact that we employed a convenience sample and that the sample included relatively few elderly men. Despite our efforts to recruit participants through verbal, printed, and online invitations and to conduct data collection at an elderly community center, this limitation remains a concern and should be carefully addressed when testing such individuals.

To our knowledge, this study provides the first S-Index prediction equations for healthy adults in Brazil. It incorporated age, weight, and height as explanatory variables, and, because of their simplicity, these equations have broad applicability in various clinical settings. 

## References

[1] Patel N, Chong K, Baydur A (2022). Methods and applications in respiratory physiology respiratory mechanics, drive and muscle function in neuromuscular and chest wall disorders. Front Physiol.

[2] Caruso P, Albuquerque ALP, Santana PV, Cardenas LZ, Ferreira JG, Prina E (2015). Diagnostic methods to assess inspiratory and expiratory muscle strength. J Bras Pneumol.

[3] Silva PE, Carvalho KL, Frazão M, Maldaner V, Daniel CR, Gomes-Neto M (2018). Assessment of Maximum Dynamic Inspiratory Pressure. Respir Care.

[4] Areias GS, Fenley A, Santiago LR, Arruda ACT, Jaenisch RB, Guizilini S (2024). Incremental Ramp Load Protocol to Assess Inspiratory Muscle Endurance in Healthy Individuals Comparison with Incremental Step Loading Protocol. Braz J Cardiovasc Surg.

[5] Kowalski T, Wilk A, Klusiewicz A, Pawliczek W, Wiecha S, Szczepanska B (2024). Reference values for respiratory muscle strength measured with the S-Index Test in well-trained athletes, e-sports athletes, and age-matched controls. Exp Physiol.

[6] Roldán A, Forte A, Monteagudo P, Cordellat A, Monferrer-Marín J, Blasco-Lafarga C (2021). Determinants of dynamic inspiratory muscle strength in healthy trained elderly. Postgrad Med.

[7] Silva FMF, Cipriano G, Lima ACGB, Andrade JML, Nakano EY, Chiappa GR (2020). Maximal Dynamic Inspiratory Pressure Evaluation in Heart Failure A Comprehensive Reliability and Agreement Study. Phys Ther.

[8] Lee KB, Kim MK, Jeong JR, Lee WH (2016). Reliability of an Electronic Inspiratory Loading Device for Assessing Pulmonary Function in Post-Stroke Patients. Med Sci Monit.

[9] Cordeiro JA, Silva CP, Britto MCA, Andrade LB (2020). Static and dynamic evaluation of respiratory muscle strength in asthmatic children and adolescents. Rev Bras Saude Mater Infant.

[10] Minahan C, Sheehan B, Doutreband R, Kirkwood T, Reeves D, Cross T (2015). Repeated-sprint cycling does not induce respiratory muscle fatigue in active adults measurements from the POWERBreathe(r) inspiratory muscle trainer. J Sports Sci Med.

[11] Areias GS, Santiago LR, Teixeira DS, Reis MS (2020). Concurrent Validity of the Static and Dynamic Measures of Inspiratory Muscle Strength Comparison between Maximal Inspiratory Pressure and S-Index. Braz J Cardiovasc Surg.

[12] Salazar-Martínez E, Gatterer H, Burtscher M, Naranjo Orellana J, Santalla A (2017). Influence of Inspiratory Muscle Training on Ventilatory Efficiency and Cycling Performance in Normoxia and Hypoxia. Front Physiol.

[13] Yañez-Sepulveda R, Alvear-Ordenes I, Tapia-Guajardo A, Verdugo-Marchese H, Cristi-Montero C, Tuesta M (2021). Inspiratory muscle training improves the swimming performance of competitive young male sprint swimmers. J Sports Med Phys Fitness.

[14] Dosbaba F, Hartman M, Batalik L, Senkyr V, Radkovcova I, Richter S (2023). A temporal examination of inspiratory muscle strength and endurance in hospitalized COVID-19 patients. Heart Lung.

[15] Romer LM, McConnell AK (2003). Specificity and reversibility of inspiratory muscle training. Med Sci Sports Exerc.

[16] Black LF, Hyatt RE (1969). Maximal respiratory pressures normal values and relationship to age and sex. Am Rev Respir Dis.

[17] Neder JA, Andreoni S, Lerario MC, Nery LE (1999). Reference values for lung function tests II. Maximal respiratory pressures and voluntary ventilation. Braz J Med Biol Res.

[18] Florindo AA, Latorre MRDO (2003). Validation and reliability of the Baecke questionnaire for the evaluation of habitual physical activity in adult men. Braz J Sports Med.

[19] Stanojevic S, Kaminsky DA, Miller MR, Thompson B, Aliverti A, Barjaktarevic I (2022). ERS/ATS technical standard on interpretive strategies for routine lung function tests. Eur Respir J.

[20] Pereira CA, Sato T, Rodrigues SC (2007). New reference values for forced spirometry in white adults in Brazil. Braz J Med Biol Res.

[21] American Thoracic Society/European Respiratory Society (2002). ATS/ERS Statement on respiratory muscle testing. Am J Respir Crit Care Med.

[22] Laveneziana P, Albuquerque A, Aliverti A (2019). ERS statement on respiratory muscle testing at rest and during exercise. Eur Respir J.

[23] Holiday DB, Ballard JE, McKeown BC (1995). PRESS-related statistics regression tools for cross-validation and case diagnostics. Med Sci Sports Exerc.

[24] Sawamura MVY, Verrastro CGY, Ferreira EVM, de Albuquerque ALP, Ribeiro SM, Auad RV (2022). Post-COVID-19 tomographic abnormalities. Int J Tuberc Lung Dis.

[25] Lafetá ML, Souza VC, Menezes TCF, Verrastro CGY, Mancuso FJ, Albuquerque ALP (2023). Exercise intolerance in post-coronavirus disease 2019 survivors after hospitalisation. ERJ Open Res.

[26] Souza RB (2002). Pressões respiratórias estáticas máximas. J Pneumol.

[27] Bland JM, Altman DG (1986). Statistical methods for assessing agreement between two methods of clinical measurement. Lancet.

[28] Dinh A, Miertschin S, Young A, Mohanty SD (2019). A data-driven approach to predicting diabetes and cardiovascular disease with machine learning. BMC Med Inform Decis Mak.

[29] Chai SS, Goh KL, Cheah WL, Chang YHR, Ng GW (2022). Hypertension prediction in adolescents using anthropometric measurements do machine learning models perform equally well?. Appl Sci.

[30] Walters J (2022). Weakness in the intensive care unit. Pract Neurol.

[31] Ricoy J, Rodríguez-Núñez N, Álvarez-Dobaño JM, Toubes ME, Riveiro V, Valdés L (2019). Diaphragmatic dysfunction. Pulmonology.

[32] Rodrigues A, Da Silva ML, Berton DC, Cipriano G, Pitta F, O'Donnell DE (2017). Maximal Inspiratory Pressure Does the Choice of Reference Values Actually Matter?. Chest.

[33] Tzanis G, Vasileiadis I, Zervakis D, Karatzanos E, Dimopoulos S, Pitsolis T (2011). Maximum inspiratory pressure, a surrogate parameter for the assessment of ICU-acquired weakness. BMC Anesthesiol.

[34] Lista-Paz A, Langer D, Barral-Fernández M, Quintela-Del-Río A, Gimeno-Santos E, Arbillaga-Etxarri A (2023). Maximal respiratory pressure reference equations in healthy adults and cut-off points for defining respiratory muscle weakness. Arch Bronconeumol.

[35] Carpenter MA, Tockman MS, Hutchinson RG, Davis CE, Heiss G (1999). Demographic and anthropometric correlates of maximum inspiratory pressure the atherosclerosis risk in communities study. Am J Respir Crit Care Med.

[36] Hepper NG, Fowler WS, Helmholz HF (1960). Relationship of height to lung volume in healthy men. Dis Chest.

[37] Neder JA, Andreoni S, Castelo-Filho A, Nery LE (1999). Reference values for lung function tests I. Static volumes. Braz J Med Biol Res.

